# Structural and functional divergence of cancer hotspot mutations in CREBBP

**DOI:** 10.1073/pnas.2605702123

**Published:** 2026-09-22

**Authors:** Wenchao Zhang, Haopeng Yang, Ariel Mechaly, Zhongyang Li, Guiling Cui, Leslie Regad, Romain Duval, Julien Dairou, Ahmed Haouz, Michael R. Green, Fernando Rodrigues Lima

**Affiliations:** ^a^ Department of Lymphoma & Myeloma, The University of Texas MD Anderson Cancer Center, Houston, TX 77030; ^b^ Institut Pasteur, Université Paris Cité, CNRS UMR 3528, Plate-forme de Cristallographie-C2RT, Paris F-75015, France; ^c^ Université Paris Cité, CNRS, Unité de Biologie Fonctionnelle et Adaptative, Paris F-75013, France; ^d^INSERM U1133, Computational Modeling of Protein-Ligand Interactions, Paris F-75013, France; ^e^Université Paris Cité and Université des Antilles, INSERM, UMRS 1134, Paris F-75015, France; ^f^ Centre National de Référence pour les Groupes Sanguins, Établissement Français du Sang Ile-de-France, Paris F-75522, France; ^g^ Université Paris Cité, CNRS UMR 8601, Laboratoire de Chimie et de Biochimie Pharmacologiques et Toxicologiques, Paris F-75006, France; ^h^ Department of Hematopoietic Biology & Malignancy, The University of Texas MD Anderson Cancer Center, Houston, TX 77030

**Keywords:** crystal structure, lysine acetyltransferase, mutation, cancer, lymphoma

## Abstract

*CREBBP* is among the most frequently mutated genes in human cancer and encodes a lysine acetyltransferase (KAT) protein that acetylates histones and nonhistone proteins to drive transcriptional activation. Mutations of *CREBBP* most frequently result in missense changes affecting the active site within the KAT domain. However, there has not yet been comprehensive biochemical, structural, and functional studies to determine the impact of recurrent KAT-domain variants. Here we show that the most frequently mutated residue of *CREBBP*, arginine 1446, alters the stability of acetyl-CoA binding within the cofactor-binding pocket but retains detectable acetyltransferase activity for a variety of substrates including histones. In contrast, other recurrently mutated residues affect the catalytic center within the KAT domain and result in a catalytically dead protein with a profound loss of histone acetylation and enhancer activity. These results highlight functionally divergent classes of *CREBBP* mutations depending on the position of the mutated residue within KAT domain.

## Significance

Epigenetic deregulation is an emerging and therapeutically targetable hallmark of cancer but the structural and functional impact of mutations in epigenetic regulators remain largely uncharacterized. Here we solve the crystal structures of recurrent hotspot mutations in the CREBBP acetyltransferase and show through biochemical and molecular assays that the arginine 1446 hotspot has a unique impact on substrate binding and acetyltransferase activity. This provides a framework with which mutations of *CREBBP* can be interpreted based upon their predicted impact on substrate binding or catalysis, linked with differences in EP300 dependence for maintenance of regulatory element activity.

Transcriptional coactivators orchestrate transcription factor (TF)-mediated gene activation by catalyzing the unpacking of chromatin or enzymatically changing the histone code through addition or removal of posttranslational modifications on histone tails and other proteins. The epigenetic regulation of transcription is frequently perturbed in cancer through somatic mutations (1), providing an attractive opportunity for therapeutic targeting (2). CREBBP (a.k.a., Cbp) and EP300 (a.k.a., p300) are structurally related acetyltransferase proteins that are thought to act as redundant transcriptional coactivators through acetylation of histones and other proteins. The *CREBBP* and *EP300* genes have both been implicated as tumor suppressor in large pan-cancer sequencing studies (3, 4), but have different mutational frequencies across cancer types that suggest a subset of nonredundant functions in different cancers.

Loss of function of *CREBBP* has been primarily modeled by gene knock-out and shown to contribute to oncogenesis (5, 6). However, *CREBBP* does not show the typical bias toward nonsense and frameshift mutations that are seen for many tumor suppressor genes (7), and missense mutations within the catalytic lysine acetyltransferase (KAT) domain have been shown to drive a greater magnitude of histone acetylation loss than knock-out (7, 8). In addition, some studies have described a subset of *CREBBP* mutations as being gain-of-function (9). To date, there has been no assessment of the structural consequences of highly recurrent *CREBBP* mutations and their relation to enzymatic activity. We therefore undertook a systematic analysis of *CREBBP* hotspot mutations to characterize their structural, biochemical, and functional consequences. We show that mutations at tyrosines 1503 and 1482, which affect the catalytic center (region within the cofactor-binding pocket, proximal to acetyl and cysteamine moieties of the cofactor in the active site of the CREBBP KAT domain), result in an inactive enzyme, whereas the most frequent hotspot mutation at arginine 1446, which is involved in acetyl-CoA (Ac-CoA) binding (through interactions with the pyrophosphate moiety in the distal part of cofactor), retains low but significant levels of activity and chromatin loading, resulting in divergent molecular phenotypes in CRISPR-edited cells.

## Results

### Mutation Spectrum of *CREBBP*.

**Fig. 1.**
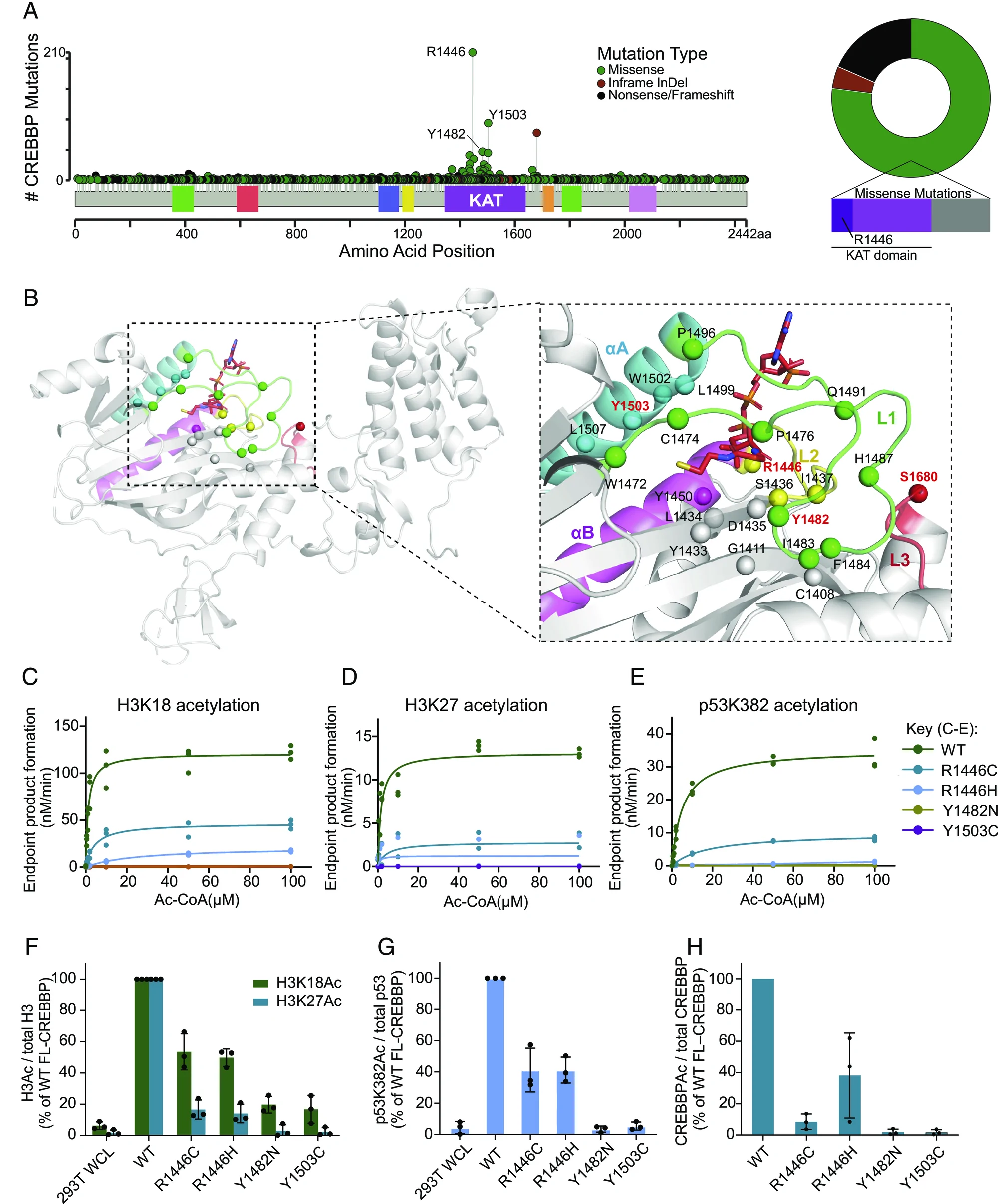
Recurrent cancer-associated CREBBP hotspot mutations differentially impair acetyltransferase activity. (*A*) Lollipop plot of somatic *CREBBP* mutations in cancer with a pie chart summarizing mutation types. (*B*) Distribution of recurrent cancer-associated CREBBP mutations mapped onto the CREBBP KAT-domain structure (shown in the CREBBP Y1503F + CoA structure; PDB ID: 8OG2 see Fig. 2 for details). Recurrently mutated residues cluster around the Ac-CoA-binding pocket and are located within or adjacent to structural elements that form the cofactor/substrate-binding pocket, including the αA helix (cyan), αB helix (magenta), L1 loop (green), L2 loop (yellow), and L3 loop (red); the remaining KAT-domain structure is shown in gray. (*C*–*E*) Fixed-time RP-UFLC endpoint acetyltransferase assays of WT and mutant CREBBP-KAT proteins using H3K18. Data show endpoint product formation in nM normalized to the 20 min incubation time (nM/min). (*C*), H3K27 (*D*), or p53K382 (*E*) peptide substrates and the indicated concentrations of Ac-CoA. Acetylated product was quantified after a 20-min incubation and expressed as nM of product formed/min. (*F* and *G*) Acetylation status of endogenous histones (*F*) and p53 (*G*) in HEK293T cells overexpressing WT or mutant CREBBP-FL, assessed by immunoblotting. (*H*) Acetylation of WT or mutant CREBBP-FL, assessed by CREBBP immunoprecipitation followed by anti-KAc immunoblotting. Signals were normalized to total H3 (*F*), total p53 (*G*), or immunoprecipitated CREBBP (*H*) and are shown relative to WT CREBBP-FL.

We first evaluated the spectrum of *CREBBP* mutations in a series of 10,478 solid tumors (3) and 4,320 non-Hodgkin lymphomas (7), collectively harboring 2,043 *CREBBP* coding mutations (Fig. 1*A*). These mutations were primarily missense variants (1,574/2,043) and enriched within the catalytic lysine acetyltransferase (KAT) domain (991/1,574) including a predominant hotspot at R1446 (210/991; Fig. 1*A*) that represented the most recurrently mutated site in both the solid tumor and lymphoma cohorts. Analysis of these cohorts revealed significant enrichment of KAT-domain mutations in both groups (*SI Appendix*, Fig. S1). Mapping mutational hotspots to CREBBP structure (Y1503F in complex with CoA, PDB ID: 8OG2) showed that they cluster within the active site, notably in the cofactor-binding pocket (Fig. 1*B*), but little inference could be drawn as to their relative functional impact on KAT activity. However, the prevalence of R1446 mutations suggests that there may be a preferential selective advantage for mutating this amino acid.

### KAT-Domain Hotspot Mutations of *CREBBP* Have Distinct Biochemical Consequences.

We explored the functional impact of the three most prevalent mutational hotspots using multiple orthogonal acetyltransferase assays with wild-type (WT) sequence, mutation of R1446 (R1446C, R1446H) or mutation of the two next most prevalent hotspots, tyrosine 1482 (Y1482N) and tyrosine 1503 (Y1503C). Reverse-phase ultra-fast liquid chromatography (RP-UFLC) with recombinant human CREBBP catalytic core (CREBBP-KAT, comprising BRD, RING, PHD, KAT, and ZZ domains) and fixed concentrations of peptide substrate (100 µM) provides quantification of the acetylated and nonacetylated product peaks over increasing concentrations of Ac-CoA that are proportional to, but not a direct measurement of, enzymatic kinetics (10). RP-UFLC showed no activity of Y1482N or Y1503C mutants and reduced but detectable activity of R1446C and R1446H mutants compared to WT for three well-characterized CREBBP substrates, histone H3 lysine 18 (H3K18), histone H3 lysine 27 (H3K27) and p53 lysine 382 (p53K382) (Fig. 1 *C*–*E*). These findings were also observed in cell-based end-point acetylation assays in which full-length human CREBBP (CREBBP-FL) was overexpressed in HEK293T cells, and acetylation of endogenous histones and p53 was assessed by immunoblotting (Fig. 1 *F* and *G* and *SI Appendix*, Fig. S2 *A* and *B*). Similar results were obtained using in vitro end-point acetyltransferase assays with standardized concentrations of Ac-CoA and recombinant protein substrates (histone H3.1 or p53) incubated with CREBBP-KAT proteins (*SI Appendix*, Fig. S2*C*) as assessed by immunoblotting (*SI Appendix*, Fig. S2 *D* and *E*). These assays additionally revealed substrate-specific differences in loss of catalytic activity for the R1446 mutants. Specifically, the activity of R1446C/H is less impaired when employing H3K18 as a substrate compared to when using H3K27 as a substrate, in line with a preference of CREBBP for H3K18 as a substrate (11). CREBBP is also able to catalyze autoacetylation, including on lysines within its autoinhibitory loop (AIL) (12). We assessed this in 293 T cells transfected with CREBBP-FL by immunoprecipitation of CREBBP followed by immunoblotting for acetyl-lysine (KAc) (Fig. 1*H* and *SI Appendix*, Fig. S2*F*). We additionally measured autoacetylation using an in vitro acetylation assay with purified CREBBP-KAT proteins that were first deacetylated with Lp-HDAC (13) and then incubated with Ac-CoA for 20 min at room temperature prior to immunoblot analysis with KAc antibody (*SI Appendix*, Fig. S2 *G* and *H*). Both approaches showed that R1446H and R1446C mutants retained reduced but variable autoacetylation activity while the Y1482N and Y1503C mutants showed no detectable autoacetylation above the background of the assay. Lower but detectable levels of acetylation were also observed with an AIL-deleted mutant of CREBBP-KAT (*SI Appendix*, Fig. S2*I*), indicating that autoacetylation can occur outside of the AIL loop. Consistent with the partial retention of R1446 mutant activity, endogenous CREBBP R1446H/C mutant alleles in RL B cell lymphoma and NCI-H82 lung cancer cells showed residual CREBBP acetyl-lysine signal and partial preservation of H3K18Ac and H3K27Ac (*SI Appendix*, Fig. S2 *J* and *K*). These biochemical assays therefore revealed differences in catalytic activity between highly recurrent *CREBBP* mutations, with the most frequent mutational hotspot (R1446) partially impairing its catalytic activity in a substrate-specific manner, and other less frequent but highly recurrent mutations (Y1482N and Y1503C) resulting in loss of any detectable catalytic activity.

### Structural Changes in the Active Site of CREBBP Lead to Altered Acetyltransferase Activity.

**Fig. 2.**
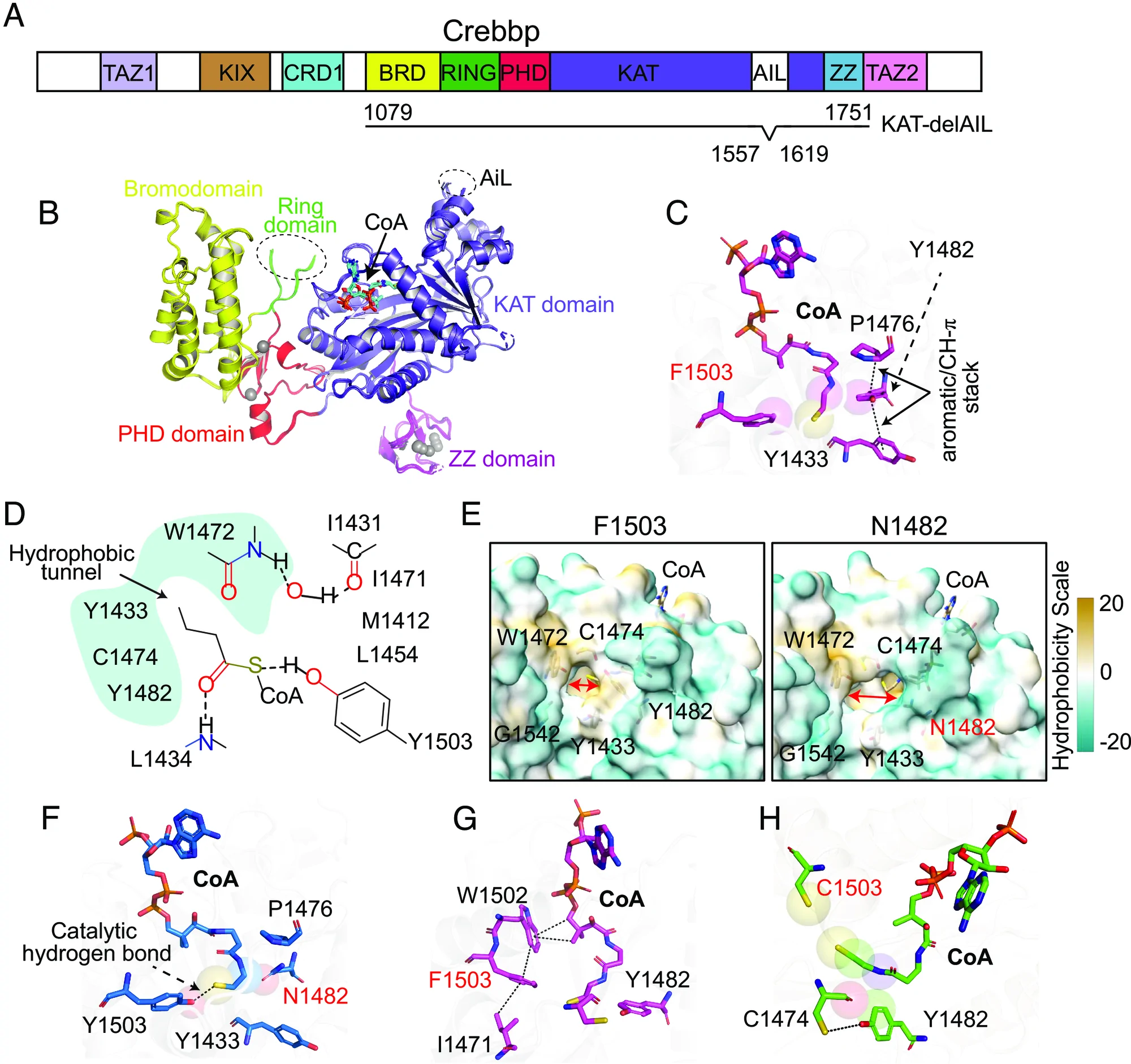
Structural analysis of the CREBBP Y1482 and Y1503 mutations. (*A*) Schematic of the CREBBP protein and CREBBP KAT-delAIL construct used for crystallographic studies. (*B*) Alignment of the structures of CREBBP mutants (Y1503F, R1446H/C, Y1482N, and Y1503C) with CoA. (*C*) Close-up of the active site highlighting the aromatic/CH-π stacking interactions involving the Y1482 in the Y1503F + CoA structure. (*D*) Hydrophobic/aromatic tunnel of CREBBP in the catalytic center (14). (*E*) Surface hydrophobicity maps of the Y1482 and N1482 structures. (*F*–*H*) The Y1503C mutation disrupts the Y1503-CoA hydrogen bond and perturbs the local interaction network, as shown in the Y1482N (*F*), Y1503F (*G*), and Y1503C (*H*) structures.

Having observed that the most highly recurrent mutations of *CREBBP* cluster around the Ac-CoA-binding pocket, and exhibit differential activity in biochemical assays, we hypothesized that these mutations may have differential effects on the structural properties of CREBBP and cofactor interactions. The KAT domain of CREBBP is highly conserved between mouse and human, including the R1446, Y1482, and Y1503 residues (*SI Appendix*, Fig. S3). Crystallization requires complete removal of acetylation and highly flexible regions and cannot be achieved easily for a fully WT protein. Prior structural studies of CREBBP and EP300 have addressed this by introduction of an inactivating Y1503F or Y1467F mutations, respectively, into the murine full-KAT domain (amino acids 1079 to 1751), in addition to removal of the flexible AIL region from the protein (14–17). We therefore followed this same approach using murine CREBBP-KAT-delAIL proteins (Fig. 2*A*). The Y1503F mutation is itself a frequent mutation observed in cancer and maintains the aromatic ring but removes the hydroxyl group. We additionally generated a Y1503C variant, which we found to be catalytically inactive and which leads to loss of the aromatic ring at this position. The Y1482N mutation was also found to be catalytically inactive and therefore did not have to be combined with the Y1503F mutation, allowing assessment of the WT configuration of Y1503 within the Y1482N structure. However, because the R1446 mutations maintained detectable activity (notably autoacetylation) in biochemical assays, they were combined with the Y1503F mutation to eliminate catalytic activity and subsequent autoacetylation. We therefore generated a suite of five variant CREBB-KATs-del-AIL (Y1503F, Y1503C, Y1482N, R1446C[+Y1503F] and R1446H[+Y1503F]) for structural analysis of recurrent KAT-domain mutations within *CREBBP*, with Y1482N serving as the control for Y1503F/Y1503C, and Y1503F serving as the control for Y1482N, R1446C, and R1446H.

High purity deacetylated proteins were produced, cocrystallized with coenzyme-A (CoA), and analyzed by X-ray crystallography, producing structures of binary complexes with 2.5 Å (Y1503F), 2.0 Å (Y1503C), 2.4 Å (Y1482N), 2.3 Å (R1446C + Y1503F), and 2.5 Å (R1446H + Y1503F) resolution (*SI Appendix*, Table S1). The overlay of the structures presented in Fig. 2*B* showed good agreement with the previously published structure of CREBBP in apo form, with RMSD values of approximately 0.5 Å (PDB ID: 5U7G; *SI Appendix*, Fig. S3) (16). The only observable differences between structures were local and found in the interaction between CREBBP and CoA, thus these interactions will be the focus. In addition, as with the Y1467F EP300 structure, which serves as a surrogate for WT EP300, the Y1503F CREBBP structure similarly represents a reliable surrogate for WT CREBBP (*SI Appendix*, Fig. S4) (18, 19).

Y1482 forms an aromatic π-π/CH-π stack with Y1433 and P1476 (Fig. 2*C*) to create a hydrophobic/aromatic substrate binding tunnel in which C1474 is positioned to form a hydrogen bond with the β-mercaptoethylamine group of CoA (Fig. 2*D*). Y1482 has been reported to reside within the hydrophobic/aromatic tunnel and to play a critical role in catalysis (15). The Y1482N mutation disrupts this aromatic/CH-π stack, slightly enlarging the cofactor-binding tunnel, and alters its hydrophobicity/aromaticity (Fig. 2 *E* and *F*), resulting in altered positioning of the β-mercaptoethylamine group within the catalytic center. Further biochemical analysis of amino acid substitutions at this position revealed that Y1482F (hydrophobic, aromatic ring), but not Y1482H (hydrophilic/polar, poor aromaticity) or Y1482L (hydrophobic, nonaromatic), retained acetyltransferase activity in vitro (*SI Appendix*, Fig. S5), indicating that both hydrophobicity and preservation of the aromatic/CH-π stack are required for catalytic activity.

Close inspection of Y1503 within the Y1482N structure revealed a hydrogen bond between the hydroxyl group of Y1503 and the terminal sulfur atom of the β-mercaptoethylamine moiety of CoA from which the acetyl group is donated (Fig. 2*F*). This H-bond has been suggested to be important for catalysis for EP300 (14) and was eliminated in the Y1503F (loss of the hydroxyl group of the side chain) (Fig. 2*G*) and Y1503C (loss of the hydroxyl group and less bulky side chain) (Fig. 2*H*) mutants contributing to an enlargement in the catalytic center of the enzyme, and a change in the positioning of the β-mercaptoethylamine moiety within the catalytic center was observed for Y1503C. Together our biochemical and structural data indicate that mutations that alter the positioning of the β-mercaptoethylamine moiety, and therefore likely the acetyl group, within the catalytic center of CREBBP (Y1503F/C, Y1482N) lead to a loss of catalytic activity.

### R1446 Mutations Alter Ac-CoA Binding While Maintaining the Catalytic Center.

**Fig. 3.**
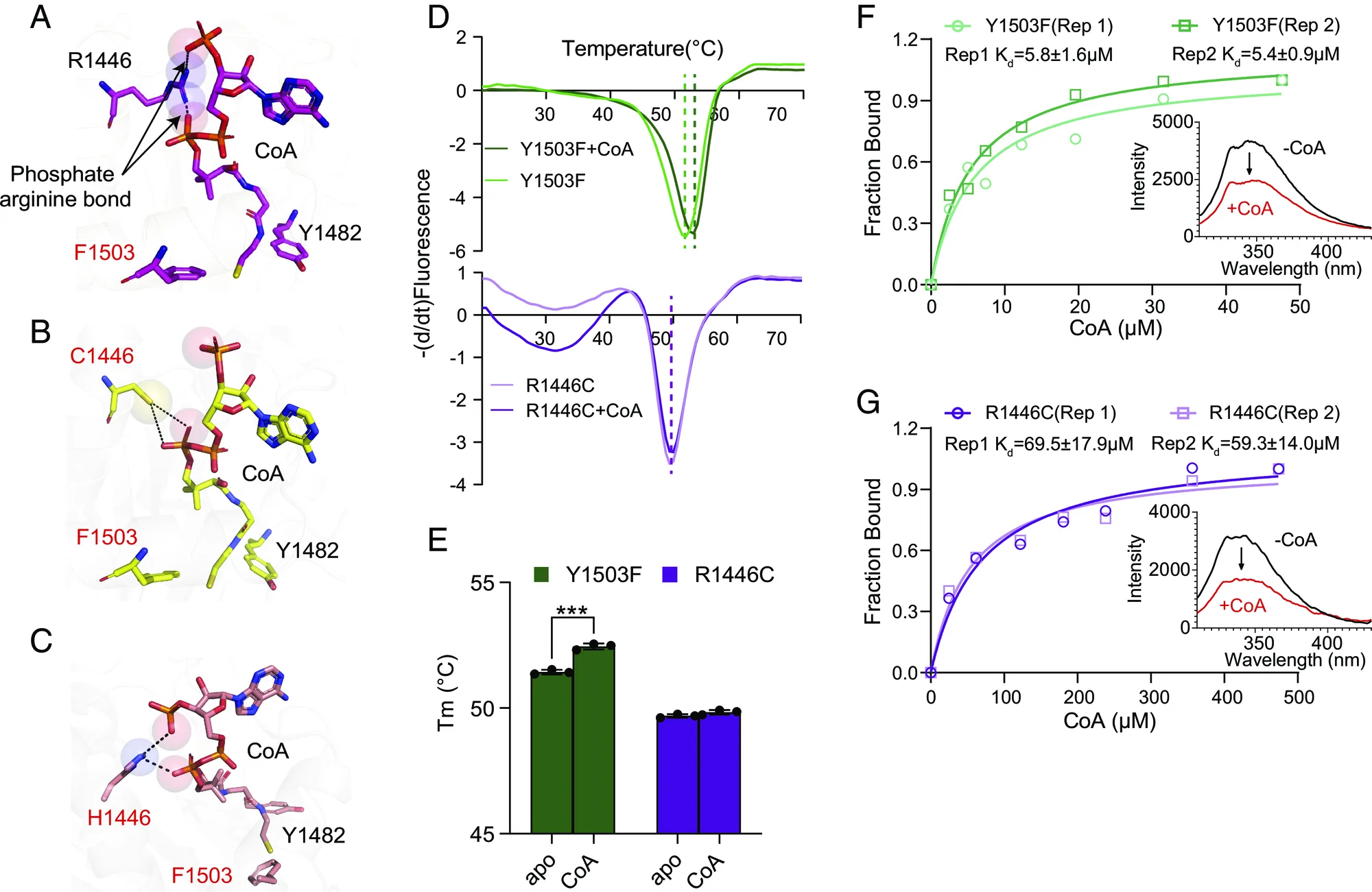
Molecular characterization of the CREBBP R1446 mutation. (*A*) Structure of the CREBBP-Y1503F active site showing a phosphate–arginine interaction between R1446 and CoA. (*B* and *C*) Loss of phosphate–arginine interaction in the structures of R1446C/H mutants cocrystallized with CoA. (*D* and *E*) Thermal shift analyses of CREBBP Y1503F and R1446C mutants incubated with or without CoA: melting curves (*D*) and derived Tm values (*E*). Two-sided paired Student’s *t* test; ***P < 0.001; unlabeled comparisons were not significant. (*F* and *G*) Fluorescence binding curves of CREBBP Y1503F (*F*) and R1446C (*G*) with CoA. The inset graphs show the decrease in intrinsic tryptophan fluorescence emission observed upon addition of the highest CoA concentration.

The R1446 residue forms a strong phosphate/arginine salt bridge with the pyrophosphate and adenosine phosphate moieties of CoA (Fig. 3*A*). The R1446C/H mutations abolish this strong interaction and enlarge the cofactor-binding pocket, resulting in modest repositioning of the pantothenic acid and adenosine diphosphate moieties, while largely preserving the position of the β-mercaptoethylamine group (Fig. 3 *B* and *C*). Electrostatic interactions between arginine side chains and phosphate groups are known to be particularly strong (20) suggesting that loss of this interaction in R1446C/H mutants substantially reduces the stability of cofactor binding. To assess the effect of this mutation on protein stability and cofactor binding, we performed thermal shift analysis (TSA) to evaluate the impact of mutation and cofactor binding on CREBBP stability. Incubation of CoA with WT CREBBP significantly increased the melting temperature (T_m_) relative to the apo protein, whereas this stabilizing effect was not observed for the R1446C mutant indicating reduced cofactor binding and consequently diminished protein stability (Fig. 3 *D* and *E*). We further assessed CoA binding using intrinsic tryptophan fluorescence. CREBBP Y1503F bound CoA with K_d_ values of 5.8 ± 1.6 and 5.4 ± 0.9 µM in two independent experiments. These values are consistent with previous studies reporting a K_d_ of approximately 7 µM for cofactor binding to p300 (14). In contrast, the R1446C mutant displayed substantially weaker CoA binding, with K_d_ values of 69.5 ± 17.9 and 59.3 ± 14.0 µM (Fig. 3 *F* and *G*). These fluorescence binding data support the conclusion that disruption of the R1446 phosphate/arginine interaction reduces cofactor binding. Consistent with this result, the Ac-CoA concentration required to achieve half-maximal endpoint product formation increased approximately threefold in the R1446 mutants (~4.5 µM) relative to WT (~1.3 µM), further indicating reduced binding efficiency (Fig. 1*C*).

Our structural analyses indicate that one class of mutations perturb the positioning of the β-mercaptoethylamine moiety, and therefore the acetyl group, within the catalytic center of CREBBP (e.g., Y1503F/C, Y1482N, W1472C) and a second class of mutations disrupt interactions with other regions of CoA cofactor (notably adenosine 3′ phosphate and pyrophosphate moieties) while maintaining the overall orientation of the β-mercaptoethylamine and acetyl group (e.g., R1446C/H, P1494A). Comprehensive profiling of a spectrum of these mutations by RP-UFLC-based acetyltransferase assays confirmed the expectation that the first class affecting the catalytic center result in complete loss of catalytic activity while the second class affecting CoA binding lead to only partial loss of catalytic activity (*SI Appendix*, Fig. S6). These data therefore support structurally informed classification of CREBBP KAT-domain mutations.

### Variable Enhancer Inactivation by Different Classes of *CREBBP* Mutations.

**Fig. 4.**
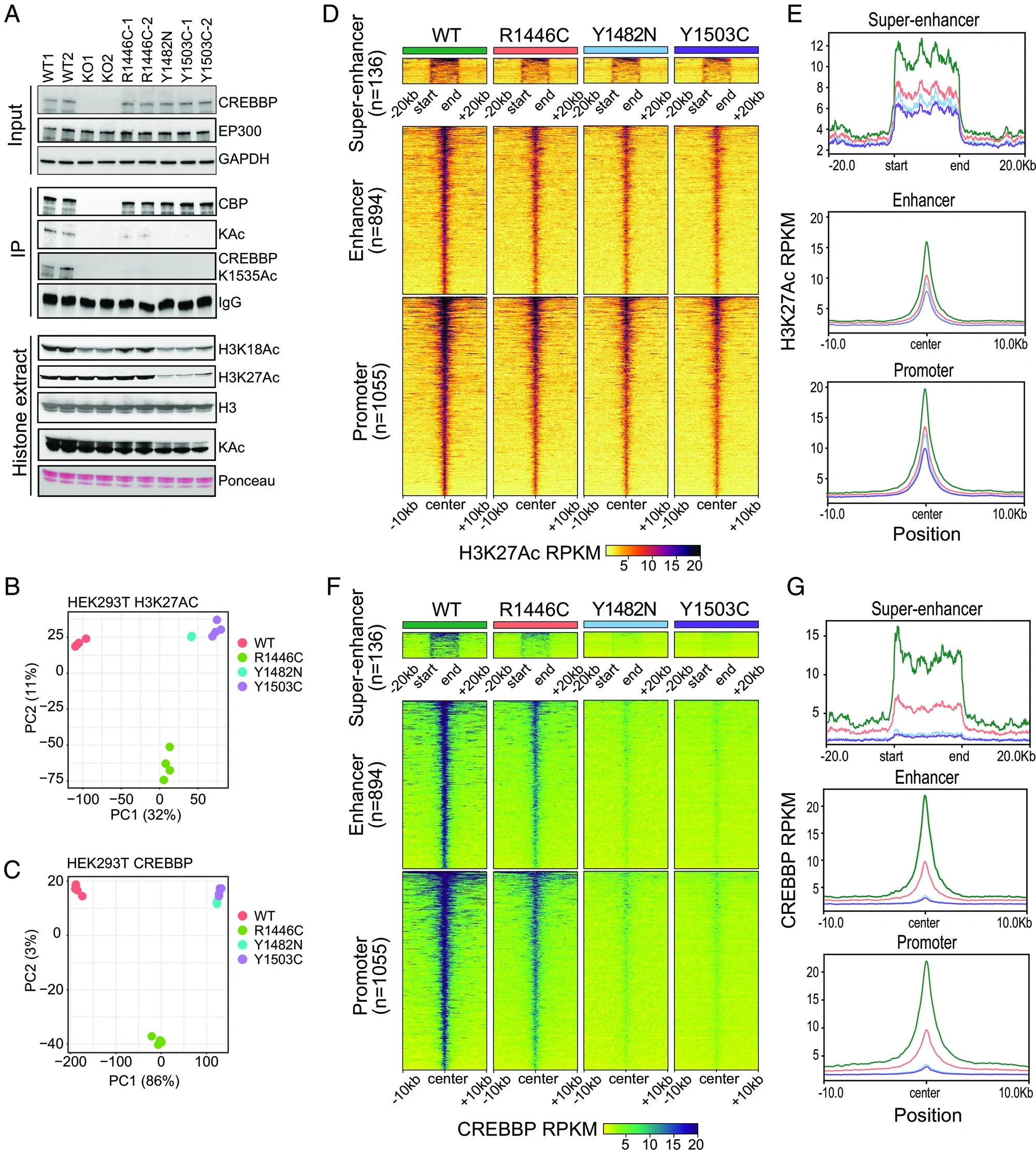
CREBBP KAT-domain mutations differentially affect histone acetylation and chromatin occupancy. (*A*) Immunoblot analysis of CREBBP expression, autoacetylation, and histone acetylation in CRISPR-edited HEK293T cells. (*B* and *C*) PCA of H3K27Ac CUT&RUN (*B*) and CREBBP CUT&RUN (*C*) data from CRISPR-edited cells. (*D* and *E*) Heat maps (*D*) and density plots (*E*) of H3K27Ac CUT&RUN in HEK293T cell line with CREBBP WT, and different KAT-PMs (R1446C, Y1482N, and Y1503C). (*F* and *G*) Heat maps (*F*) and density plots (*G*) of CREBBP CUT&RUN in HEK293T cell line with CREBBP WT, and different KAT-PMs (R1446C, Y1482N, and Y1503C).

CREBBP is recruited to chromatin by transcription factors as a coactivator and acetylates histone tails to drive transcriptional activation. We have previously observed that KAT-domain mutations of CREBBP lead to unloading from chromatin and loss of H3K27 acetylation (H3K27Ac) at enhancers that are dynamically loaded and unloaded in B-cells (7). However, the effect of KAT mutations in other cellular contexts has not been explored. We therefore compared the effect of R1446C, Y1482N, and Y1503C mutants to WT *CREBBP* in 293 T cells by CRISPR editing the endogenous sequence and performing CUT&RUN for CREBBP loading and H3K27Ac. All KAT mutations retained expression of CREBBP protein but had variable reductions in autoacetylation assessed by immunoprecipitation of CREBBP followed by immunoblotting for KAc or CREBBP K1535Ac, and in histone acetylation by immunoblotting of histone extracts (Fig. 4*A*). Principal component analysis (PCA) of H3K27Ac and CREBBP CUT&RUN data showed clear separation of mutant and WT clones, and separation of R1446C mutants and Y1482N/Y1503C mutants suggesting that differences in activity are associated with divergent enhancer loading and activation (Fig. 4 *B* and *C*). Differential analysis of KAT mutants to WT identified hundreds of peaks with loss of H3K27Ac at superenhancers, enhancers, and promoters (Fig. 4*D* and Dataset S2), all of which showed the greatest loss in Y1503C mutants, the least magnitude of loss in R1446C mutants, and an intermediate level of loss in the single Y1482N mutant clone (Fig. 4*E*). Evaluation of CREBBP chromatin loading by CUT&RUN showed that R1446C mutants retain reduced but detectable levels of CREBBP loading over regions with reduced H3K27Ac (Fig. 4*F*), while the Y1482N and Y1503C mutants have almost complete loss of chromatin loading over these regions that approaches the background of the assay (Fig. 4 *F* and *G*). Histone acetylation over these regions is likely maintained by EP300, which shows reduced but detectable levels of chromatin loading over these regions in all mutant conditions (*SI Appendix*, Fig. S7 *A* and *B*). RNA-seq analysis showed transcriptional separation by PCA, and GSEA revealed significant depletion of enhancer-loss-associated genes in the catalytically dead mutant but not in R1446C (*SI Appendix*, Fig. S7 *C* and *D*). Structurally linked differences in catalytic activity between CREBBP mutants are therefore associated with divergent patterns of chromatin loading and histone acetylation at regulatory elements.

## Discussion

In the postgenomic era of cancer research, with tens of thousands of tumors having been DNA sequenced, we can begin to appreciate nuances within the mutational spectra of cancer driver genes. Discerning functional differences between mutational hotspots may have important implications for their therapeutic targeting and provide broader biological insights into how proteins function. Here we have performed comprehensive structural, biochemical, and functional analysis of mutational hotspots within the catalytic domain of the tumor suppressor gene, *CREBBP*.

Cancer-associated *CREBBP* alterations also occur outside the KAT domain and may perturb CREBBP function through mechanisms other than direct impairment of catalysis. For example, mutations or truncations affecting TAZ2 domain can relieve intramolecular autoinhibition and enhance p300/CBP acetyltransferase activity (21). Therefore, characterization of non-KAT regulatory alterations will be important for defining the full spectrum of molecular and functional consequences associated with *CREBBP* mutations in cancer.

In both solid tumors and lymphomas, the most prominent mutational hotspot is at R1446 which we found to retain low but detectable levels of acetyltransferase activity in comparison to other mutational hotspots that are catalytically dead. These differences in catalytic activity are related to the structural impact of each mutation, with R1446 mutations reducing the stability of CoA binding while other mutations such as Y1482 and Y1503 directly impact the shape of catalytic center, positioning of cofactor (cysteamine moiety and, likely, acetyl group) and catalysis. The prevalence of R1446 mutations suggests that there may be a selective advantage toward retaining limited catalytic activity of CREBBP. This may be related to the retention of chromatin loading and enhancer activity with this mutant compared to catalytically dead mutants, suggestive of a “Goldilocks effect” related to enhancer activity in cancer.

EP300 has been identified as a potential therapeutic dependency in *CREBBP* mutant tumors (22, 23) and EP300-selective inhibitors and degraders are currently in development (24). EP300 residues corresponding to the recurrent CREBBP hotspots R1446, Y1482, and Y1503 (namely EP300 R1410, Y1446, and Y1467) are also recurrently mutated in cancer, supporting the possibility that related mutational mechanisms may occur in both proteins (Dataset S1). In line with this, we observed EP300 loading at enhancers and promoters with reduced H3K27Ac in CREBBP mutant cells, suggesting that EP300 has a role in maintaining reduced activity of these regulatory elements. However, the retention of R1446 mutant CREBBP on chromatin and higher levels of H3K27Ac in these cells suggest that cells bearing R1446 mutations may be less dependent upon EP300 to maintain regulatory element function than other mutants which lead to a complete loss of CREBBP activity and chromatin loading. It remains to be determined whether these differences in CREBBP function across mutants or mutational classes will affect the therapeutic window for EP300-targeted agents in *CREBBP* mutant tumors. Our data suggest that it may be necessary to evaluate these agents across multiple *CREBBP* mutations with different levels of activity.

Our study identified the structural basis for functional differences between classes of *CREBBP* mutations, including a retention of chromatin loading and catalytic activity of the most frequent hotspot mutation of *CREBBP*, R1446. The retained activity of this mutant may impact relative contribution of EP300 to enhancer maintenance and therefore the dependence of R1446-mutant tumors on EP300. Together, these structural, biochemical, and functional analyses of recurrent mutations in *CREBBP* shed light onto CREBBP function. In addition to providing a structural basis for understanding cofactor binding, our results indicate that while these mutations do not induce global structural disruption, the most prevalent cancer-associated alterations (affecting residues R1446, Y1482, and Y1503) specifically remodel the active site, including the cofactor-binding pocket and catalytic center. This results in divergent enzymatic consequences, ranging from complete loss of acetyltransferase activity to retention of reduced yet functionally significant activity.

In conclusion, comprehensive structural, biochemical, and molecular analysis has identified divergent effects of cancer hotspot mutations in *CREBBP*, allowing insights into the substrate binding and acetyltransferase function of CREBBP with implications for cancer biology and therapeutic targeting.

## Materials and Methods

### CREBBP Mutation Data.

Data for CREBBP mutations were compiled from published lymphoma cohorts (7) and CREBBP variants identified in solid tumors from the Genomics England 100,000 Genomes Project (100kGP; version 19, released 31 October 2024) (25). All variants were standardized to hg38 genomic coordinates and visualized using MutationMapper in cBioPortal.

### Endpoint RP-UFLC-Based Peptide Acetyltransferase Assay.

CREBBP-catalyzed peptide acetylation was measured using a fixed-time endpoint assay and quantified by RP-UFLC on a Shimadzu system, as previously described (10). Reactions (100 µL) contained 25 mM Tris-HCl (pH 8.0), 1 mM DTT, Ac-CoA as indicated (0 to 100 µM or fixed at 100 µM), 100 µM peptide substrate (H3K18, H3K27, or p53; ProteoGenix), and purified human CREBBP catalytic core (CREBBP-KAT, residues 1,079 to 1,751). Following incubation for 20 min at room temperature, reactions were quenched with 15% HClO_4_ and analyzed on a Shim-pack XR-ODS column (40 °C) using isocratic elution (80% acetonitrile, 0.12% TFA; 0.6 mL min^−1^). Under these conditions, acetylated and nonacetylated peptides were resolved as distinct peaks. The acetylated product peak was identified using the corresponding synthetic acetylated peptide standard and quantified by peak-area integration. Product concentration was calculated from calibration curves generated using known concentrations of synthetic acetylated peptide standards, normalized to the fixed 20-min incubation time, and reported as endpoint product formation.

### In Vitro Endpoint Acetyltransferase Assays Using Recombinant Histone H3.1 or p53.

Purified CREBBP-KAT (100 ng) was incubated with recombinant histone H3.1 or p53 (500 ng) in the presence of 100 µM Ac-CoA for 20 min at room temperature. Reactions were stopped by the addition of Laemmli buffer, resolved by SDS-PAGE, and analyzed by immunoblotting using antibodies against H3K18Ac (CST #9675S), H3K27Ac (Abcam ab4729), p53K382Ac (CST #2525), or KAc (CST #9441). Acetylation signals were quantified and expressed relative to the indicated control.

### Cellular Histone H3, p53, and CREBBP Acetylation Status.

The acetylation status of histone H3, p53, and CREBBP was assessed in transiently transfected or CRISPR-edited cells. Histone extracts were analyzed by immunoblotting using antibodies against H3K18Ac, H3K27Ac, and total histone H3. Whole-cell extracts were analyzed by immunoblotting using antibodies against p53K382Ac, p53, and GAPDH. To assess CREBBP acetylation, CREBBP was immunoprecipitated from whole-cell extracts and analyzed by immunoblotting using antibodies against KAc, CREBBP K1535Ac, and CREBBP. H3K18Ac and H3K27Ac signals were normalized to total histone H3; p53K382Ac signals were normalized to total p53; and KAc following CREBBP immunoprecipitation was normalized to immunoprecipitated CREBBP.

### Protein Expression and Purification.

cDNAs encoding human CREBBP-KAT, mouse CREBBP fragments (residues 1,079 to 1,751, with AIL loop residues 1,557 to 1,618 replaced by a 5-aa SGGSG linker) (2) or CREBBP-AIL loop were subcloned into pET28a (Addgene, 69864-3). Constructs were transformed into BL21-CodonPlus (DE3)-RIL cells (Agilent, 230245) for recombinant protein expression. Cells were grown in LB medium (Millipore Sigma, L3522) supplemented with kanamycin (30 µg/mL; Sigma-Aldrich, K4000) and chloramphenicol (20 µg/mL; Sigma-Aldrich, C0378) and induced with 0.5 mM IPTG (Millipore Sigma, I6758) at OD600 0.6 to 0.8. Cultures were incubated overnight at 16 °C and harvested by centrifugation. Cell pellets were resuspended in lysis buffer (25 mM Tris-HCl pH 8.0, 300 mM NaCl, 5% Triton X-100, 1 mM DTT, 1 mg/mL lysozyme), incubated on ice, and sonicated. Clarified lysates were incubated with His-Select Nickel Affinity Gel (Millipore Sigma, P6611) containing 5 mM imidazole (Millipore Sigma, 56750). Resin was washed with 10 mM imidazole, and proteins were eluted with 300 mM imidazole. Buffer exchange was performed using PD-10 desalting columns (Cytiva, 17043501) to remove imidazole. Purified proteins were used immediately or flash-frozen and stored at −80 °C.

### Protein Crystallization.

Crystallization screening trials were carried out by the vapor diffusion method using a Mosquito TM nanodispensing system (STPLabtech, Melbourn, UK) following established protocols (26) with CREBBP-KAT-delAIL proteins (10 mg/mL) in presence of CoA (1 mM). Initial hits were reproduced by hanging-drop vapor diffusion at 18 °C. The best crystals were obtained by mixing each complex with reservoir solution containing 0.3 M trisodium citrate and 20% (w/v) PEG3350 at 1:1, 1:2, or 2:1 ratios. Crystals were cryoprotected with reservoir solution supplemented with 25% (v/v) ethylene glycol and flash-cooled in liquid nitrogen.

### Diffraction Data Collection and Structure Determination.

X-ray diffraction data were collected at beamlines PROXIMA 1 and PROXIMA 2a (Synchrotron SOLEIL, St. Aubin, France) and processed with autoPROC (27). The crystal structures were solved by molecular replacement using Phaser (28). The final models were obtained through iterative cycles of manual model building with Coot (29) and reciprocal space refinement with Buster (30) and Phenix (31) Coordinates and structure factors have been deposited in the Protein Data Bank under accession codes 8CNB, 8CN0, 8CMZ, 8OG2, and 9TZ6. Structural analysis and visualization were carried out using PyMOL (32) or UCSF ChimeraX (33).

### CRISPR Gene Editing and CUT&RUN.

CRISPR editing of CREBBP and CUT&RUN analysis of H3K27Ac and CREBBP was performed as previously described (7). Briefly, HEK293T cells were electroporated using the Alt-R CRISPR-Cas9 system (Integrated DNA Technologies) with SpCas9 HiFi nuclease (IDT, 1081061), ATTO550-labeled guide RNA (IDT, 1075928), and a single-stranded oligonucleotide donor template. Forty-eight hours postelectroporation, single ATTO550-positive cells were sorted into 96-well plates, clonally expanded, and validated by Sanger sequencing of PCR amplicons spanning the targeted loci. CUT&RUN was performed using antibodies against CREBBP (Abcam, ab10490), EP300 (Bethyl, A300-358A), H3K27Ac (Thermo Fisher Scientific, MA5-23516), or IgG isotype controls (EpiCypher, 13-0042).

### Statistics.

Data are presented as mean ± SD from three independent experiments. Statistical analyses were performed using GraphPad Prism 9. Densitometric analysis of immunoblots analysis was performed using Image-Pro Plus 6.0.

### Data Access Statement.

The CUT&RUN and RNA-seq data generated in this study have been deposited in the Gene Expression Omnibus under accession number GSE337323.

Data from the National Genomic Research Library (NGRL) used in this research are available within the secure Genomics England Research Environment. Access to NGRL data is restricted to adhere to consent requirements and protect participant privacy. Data used in this research include cancer-associated variant data from the Genomics England 100,000 Genomes Project, version 19, released 31 October 2024. CREBBP variants were retrieved from the cancer_tier_and_domain_variants table accessed via LabKey. Derived datasets include filtered CREBBP variant and mutation-summary tables used in this manuscript. No participant identifiers or information restricted under the Airlock Policy are included.

Access to NGRL data is provided to approved researchers who are members of the Genomics England Research Network, subject to institutional access agreements and research project approval under participant-led governance. For more information on data access, visit: https://www.genomicsengland.co.uk/research.

## Supplementary Material

Appendix 01 (PDF)

Dataset S01 (XLSX)

Dataset S02 (XLSX)

## Data Availability

Crystal structure data have been deposited in Protein data bank. The CUT&RUN and RNA-seq data generated in this study have been deposited in the Gene Expression Omnibus under accession number GSE337323 (34). Data from the National Genomic Research Library (NGRL) used in this research are available within the secure Genomics England Research Environment. Access to NGRL data is restricted to adhere to consent requirements and protect participant privacy. Data used in this research include cancer-associated variant data from the Genomics England 100,000 Genomes Project, version 19, released 31 October 2024. CREBBP variants were retrieved from the cancer_tier_and_domain_variants table accessed via LabKey. Derived datasets include filtered CREBBP variant and mutation-summary tables used in this manuscript. No participant identifiers or information restricted under the Airlock Policy are included. Access to NGRL data is provided to approved researchers who are members of the Genomics England Research Network, subject to institutional access agreements and research project approval under participant-led governance. For more information on data access, visit: https://www.genomicsengland.co.uk/research (35) (Multiple reports attached as supporting information).
