# Supplementary material for: Structural and functional divergence of cancer hotspot mutations in CREBBP
Source: Proc Natl Acad Sci U S A. 2026 Sep 22;123(39):e2605702123. doi: 10.1073/pnas.2605702123 (PMC13624666; doi:10.1073/pnas.2605702123)
Supplement: Supplementary file 1 [file pnas.2605702123.sapp.pdf]

Supporting Information for

Structural and functional divergence of cancer hotspot mutations in CREBBP

Wenchao Zhang[1]*, Haopeng Yang[1]*, Ariel Mechaly[2], Zhongyang Li[3], Guiling Cui[3], Leslie Regad[3,4], Romain Duval[5,6], Julien Dairou[7], Ahmed Haouz[2], Michael R. Green[1,8§], Fernando Rodrigues Lima[3§]

[1]Department of Lymphoma & Myeloma, The University of Texas MD Anderson Cancer Center, Houston, TX, USA; [2]Institut Pasteur, Université Paris Cité, CNRS UMR 3528, Plate-forme de Cristallographie-C2RT, F-75015, Paris, France; [3]Université Paris Cité, CNRS, Unité de Biologie Fonctionnelle et Adaptative, F-75013 Paris, France; [4]INSERM U1133, CMPLI, F-75013 Paris, France; [5]Université Paris Cité and Université des Antilles, INSERM, UMRS 1134, F-75015 Paris, France; [6]Centre National de Référence pour les Groupes Sanguins, Établissement Français du Sang Ile-de-France, Paris, France; [7]Université Paris Cité, CNRS UMR UMR 8601, Laboratoire de Chimie et de Biochimie Pharmacologiques et Toxicologiques, F-75006, Paris, France; [8]Department of Hematopoietic Biology & Malignancy, The University of Texas MD Anderson Cancer Center, Houston, TX, USA.

§Correspondence to Fernando Rodrigues Lima or Michael R. Green

*W.Z. and H.Y. contributed equally to this study.

Email: fernando.rodrigues-lima@u-paris.fr; mgreen5@mdanderson.org

This PDF file includes:

Supplementary Methods
Figures S1 to S7
Table S1
Supplementary References

Supplementary Methods

Cell Culture and Transfection

Both parental cell lines and CRISPR-modified RL and NCI-H82 cell lines were cultured in RPMI medium with 10% FBS and 1× penicillin/streptomycin. HEK293T cells were cultured in DMEM medium with 10% FBS and 1× penicillin/streptomycin. Cells were seeded in 10 cm dishes and transfected at approximately 70% confluency with 6 µg FLAG-tagged human CREBBP plasmid (OriGene, RC219036) using METAFECTENE PRO transfection reagent (Biontex, T020). DNA and transfection reagent were complexed for 20 min at room temperature before addition to cells. After 48 h, cells were harvested and lysed; supernatants were used for immunoprecipitation and immunoblotting, and cell pellets were used for histone extraction.

Immunoprecipitation and Histone Acid Extraction

Cells were lysed in PBS (pH 7.5) containing 1% Triton X-100 (Sigma-Aldrich, X100RS), 3.3 µM trichostatin A (TSA) (Sigma-Aldrich, T8552), 10 mM sodium butyrate (Sigma-Aldrich, 303410), 1 mM DTT, and protease inhibitors (Roche, 11836170001) for 30 min on ice. Lysates were clarified by centrifugation (15,000 × g, 15 min, 4 °C), and protein concentration was determined by the Bradford assay (BioRAD, 5000201).

For immunoprecipitation, 1 mg of total protein from clarified cell lysates was incubated overnight at 4 °C with 1 µg anti-CREBBP (Santa Cruz, A22) or anti-FLAG (OriGene, TA50011). Protein A agarose beads (Santa Cruz, sc-2001) were added and incubated for 2 h at 4 °C. Beads were washed three times with lysis buffer, and bound proteins were eluted by boiling in 2× Laemmli buffer containing 200 mM β-mercaptoethanol for 5 min at 100 °C. Eluted proteins were resolved by SDS-PAGE and analyzed by immunoblotting.

For histone extraction, cell pellet was incubated overnight at 4 °C in 200 µL 0.2 N HCl and centrifuged at 6,500 × g for 10 min. The supernatant, containing acid-extracted histones, was collected, and 1 µg of histone proteins was resolved by 18% SDS-PAGE for immunoblotting.

Immunoblotting

Proteins were separated by SDS-PAGE and transferred to nitrocellulose (0.45 µm; GE Healthcare) for 2 h at 4 °C. Membranes were blocked in 5% non-fat milk in PBST for 2 h, incubated with primary antibodies in 1% milk/PBST overnight at 4 °C with agitation, washed three times in PBST, and incubated with HRP-conjugated secondary antibodies in 1% milk/PBST for 1 h at RT. Signals were developed with Clarity Western ECL (Bio-Rad, 1705061) and imaged on a ChemiDoc MP (Bio-Rad).

Site-Directed Mutagenesis

Point mutations were introduced into the CREBBP coding sequence using the QuikChange II XL kit (Agilent Technologies, 200522). Primers were designed with the Agilent QuikChange Primer Design tool. Constructs included CREBBP-KAT and FLAG-tagged CREBBP-FL for activity assays, and CREBBP-KAT-delAIL for crystallization.

Thermal Shift Assay

Thermal stability of CREBBP-KAT-delAIL Y1503F and Y1503F/R1446C was assessed by Thermal Shift Assay. Protein (1.5 µM) was mixed with 10× SYPRO Orange (Sigma, S5692) in 10 µL of buffer (25 mM Tris-HCl, pH 8.0, 300 mM NaCl) and incubated for 10 min at RT in 384-well opaque PCR plates (Roche, 04729749001). Plates were centrifuged (1,300 rpm, 4 °C) and fluorescence (excitation 465 nm, emission 580 nm) was recorded on a LightCycler® 480 System (Roche).

Intrinsic Tryptophan Fluorescence Quenching Assays

CoA binding to purified CREBBP-KAT-delAIL proteins was measured by intrinsic tryptophan fluorescence spectroscopy at 25 °C using an approach adapted from Liu et al. (1). Briefly, fluorescence spectra were recorded on a Hitachi F-7000 fluorescence spectrophotometer equipped with FL Solutions software. Measurements were performed in 50 mM HEPES, 50 mM NaCl, and 1 mM DTT (pH 8.0) using 200 nM protein in a final volume of 400 µL in a Hellma high-precision quartz fluorescence cuvette. Intrinsic

tryptophan fluorescence was excited at 295 nm, and emission spectra were collected from 310 to 430 nm. Excitation and emission slit widths were set to 2.5 and 5.0 nm, respectively. CoA was added stepwise to the protein solution and mixed before each measurement. Final CoA concentrations were 0, 2.5, 5.0, 7.4, 12.3, 19.6, 31.5, and 47.6 μM for Y1503F, and 0, 24.9, 61.7, 122.0, 180.7, 238.1, 356.3, and 473.9 μM for Y1503F/R1446C. Following each addition, samples were allowed to equilibrate prior to data acquisition. Fluorescence spectra were corrected by subtracting the corresponding buffer background. CoA binding was monitored by quenching of intrinsic tryptophan fluorescence and quantified from the fluorescence intensity at the emission maximum for each titration series. Fluorescence changes were normalized to fractional occupancy (fraction bound) and fitted using the quadratic binding equation to estimate the dissociation constant K_d (1). Independent experiments were fitted separately, and K_d values are reported for each replicate.

RNA-seq

Total RNA was isolated with the RNeasy Micro Kit (Qiagen, 74004) with on-column DNase treatment, according to the manufacturer's instructions. RNA-seq libraries were prepared from 500 ng of RNA using KAPA HyperPrep RNA with the RiboErase (Roche KK8560) kit. Libraries were validated on TapeStation 4200, quantified with Qubit High-Sensitivity dsDNA, multiplexed, and sequenced on a NovaSeq 6000 (100-10-10-100 bp).

Data Processing

CUT&RUN processing was performed as previously described (2). Sequencing quality control was evaluated by FastQC. Paired-end raw reads were trimmed to remove adapter sequences using Trimmomatic and then aligned to version 19 of the human reference genome (hg19) using Bowtie2 with options: --local --very-sensitive --no-mixed --no-discordant --phred33 -I 10 -X 700. Aligned reads were sorted by coordinates and tested for mapped fragment size distribution by Samtools. The final aligned and sorted Bam files were used for further processing. Visualization was assessed by converting the final Bam files to Bigwig files using the 'bamCoverage' command in deepTools with option: --normalizeUsing RPKM -e -bs 50 and evaluated in IGV browser (Broad Institute). Peaks were called by MACS2 with option: -g hs -B -f BAM -q 0.01. IgG controls were used for comparison. Each technical repeat and biological repeat were separately processed.

RNA-seq processing was performed as previously described (2). Reads were trimmed with Trimmomatic and quality-checked with FastQC before and after trimming. Trimmed reads were aligned to hg19 with STAR. Samples with ≥20 million uniquely mapped reads were retained. Read counting was performed with featureCounts (Subread). Rows with fewer than 10 total reads were filtered prior to differential expression analysis with DESeq2 in R. Principal components were computed with DESeq2 and visualized with ggplot2. A gene set enrichment analysis (GSEA) was performed using GSEA software.

Figures

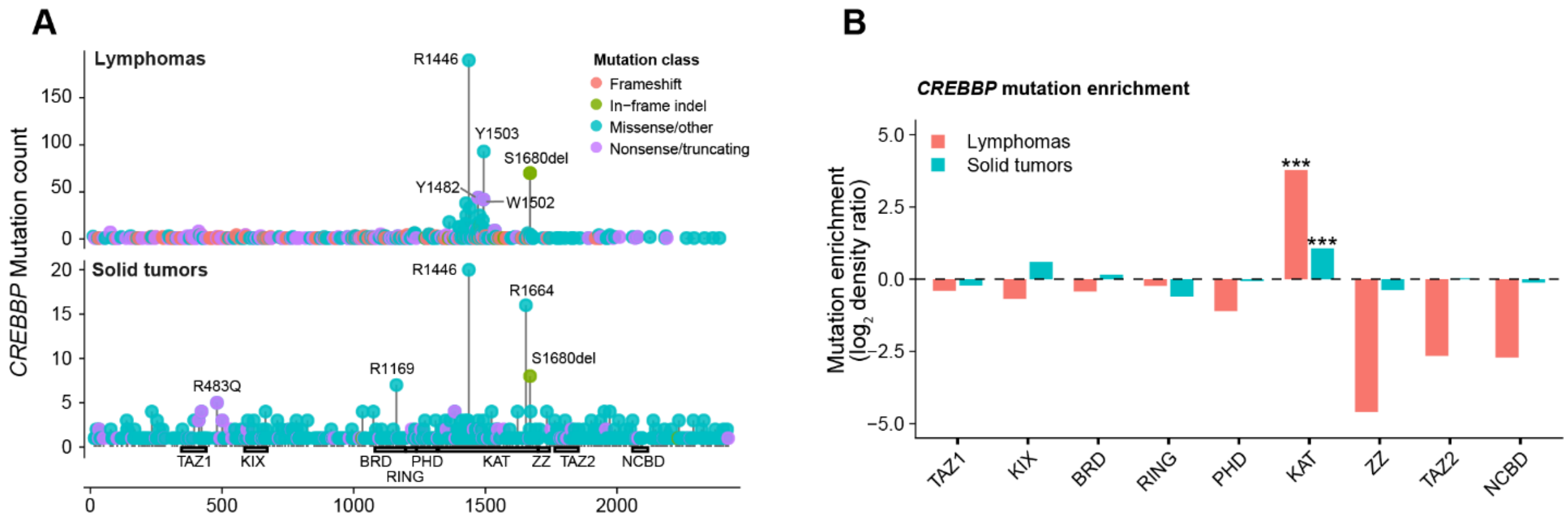

Fig. S1. *CREBBP* mutation spectrum and domain enrichment. (A) *CREBBP* mutations in lymphoma and solid tumor cohorts were mapped across the protein sequence and colored by mutation class. (B) Domain-level enrichment of *CREBBP* mutations. Bars indicate the log_2 mutation density ratio within each domain versus the remaining protein regions. Significance was assessed by Fisher's exact test with Benjamini–Hochberg FDR correction. ***FDR-adjusted $P < 0.001$.

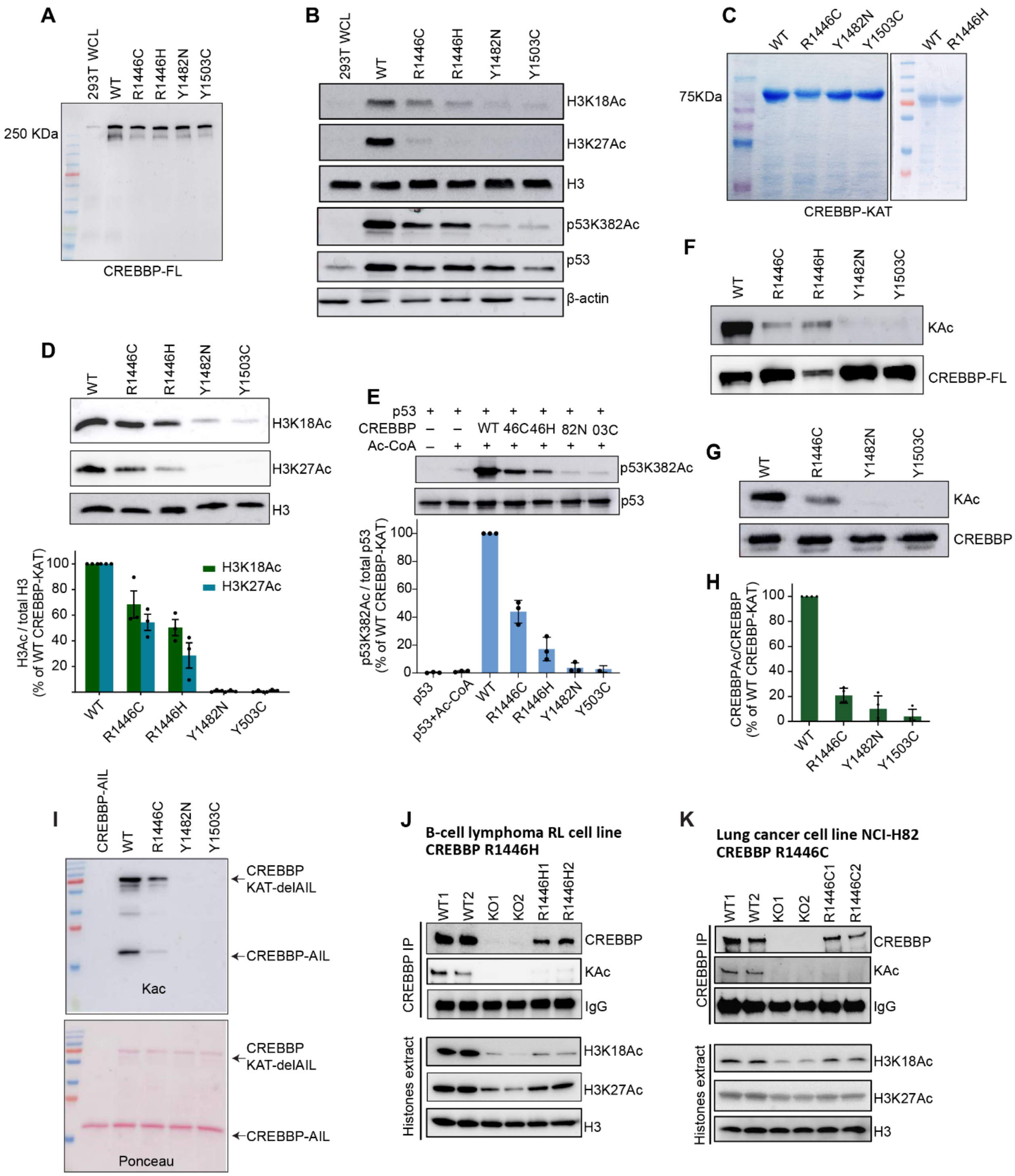

Fig. S2. Effects of recurrent CREBBP KAT-domain hotspot mutations on acetyltransferase activity. (A) Immunoblot analysis showing overexpression of CREBBP-FL (WT and mutants) in HEK293T cells. (B) Acetylation of endogenous histone H3 (H3K18Ac and H3K27Ac) and p53 (p53K382Ac) in HEK293T cells overexpressing CREBBP-FL, assessed by immunoblotting. (C) Coomassie-stained SDS-PAGE of purified CREBBP-KAT proteins (WT and mutants). (D-E) *In vitro* acetyltransferase activity of CREBBP-KAT toward recombinant histone H3 (D) and p53 (E), detected by immunoblotting. (F) Autoacetylation of transfected CREBBP-FL detected by immunoprecipitation followed by immunoblotting for KAc. (G-H) Purified WT and mutant CREBBP-KAT proteins were first deacetylated with *Legionella pneumophila* HDAC (Lp-HDAC), then subjected to an *in vitro* autoacetylation assay. Autoacetylation was assessed by immunoblotting with

the KAc antibody (G) and quantified relative to total CREBBP protein (H). (I) CREBBP-KATΔAiL-mediated acetylation of the CREBBP-AIL loop *in vitro*. Lp-HDAC-treated CREBBP-KAT-delAIL was incubated with CREBBP-AIL in the presence of Ac-CoA for 20 min, and acetylation was assessed by KAc immunoblotting. Ponceau staining indicates protein loading. (J, K) CRISPR-edited cancer cell models harboring endogenous *CREBBP* R1446 mutations were analyzed for CREBBP autoacetylation and histone acetylation. RL B-cell lymphoma cells harboring CREBBP R1446H (J) and NCI-H82 lung cancer cells harboring CREBBP R1446C (K) were subjected to CREBBP immunoprecipitation followed by immunoblotting for total CREBBP and KAc. Histone extracts were analyzed by immunoblotting for H3K18Ac, H3K27Ac, and total H3.

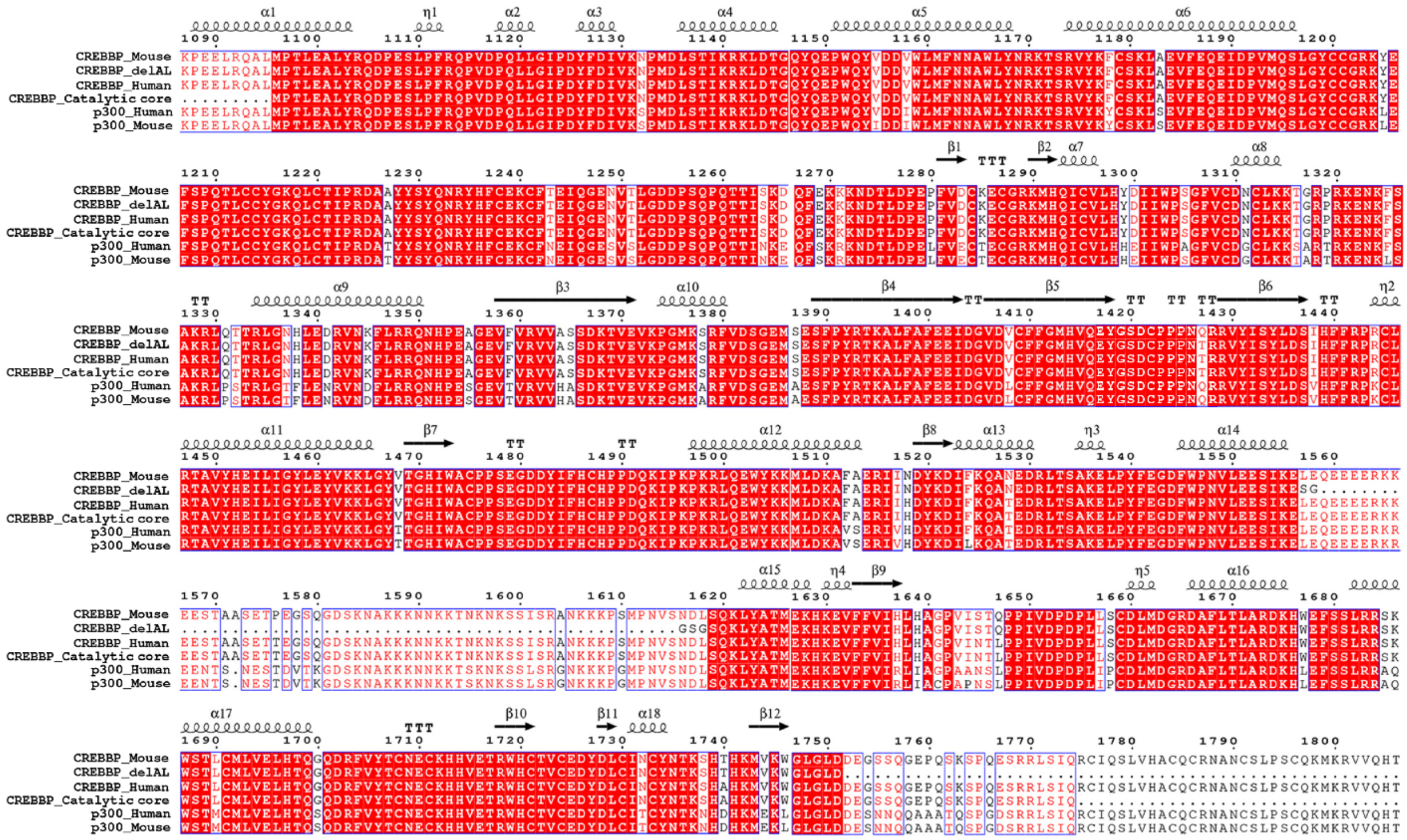

Fig. S3. Sequence alignment of CREBBP and EP300 catalytic domains. Sequences were retrieved from UniProt/NCBI RefSeq as follows: CREBBP (human, UniProt Q92793); CREBBP (mouse, NCBI RefSeq NM_001025432); EP300 (human, UniProt Q09472); EP300 (mouse, UniProt B2RWS6). The multiple-sequence alignment was computed with ClustalW and visualized with ESPript.

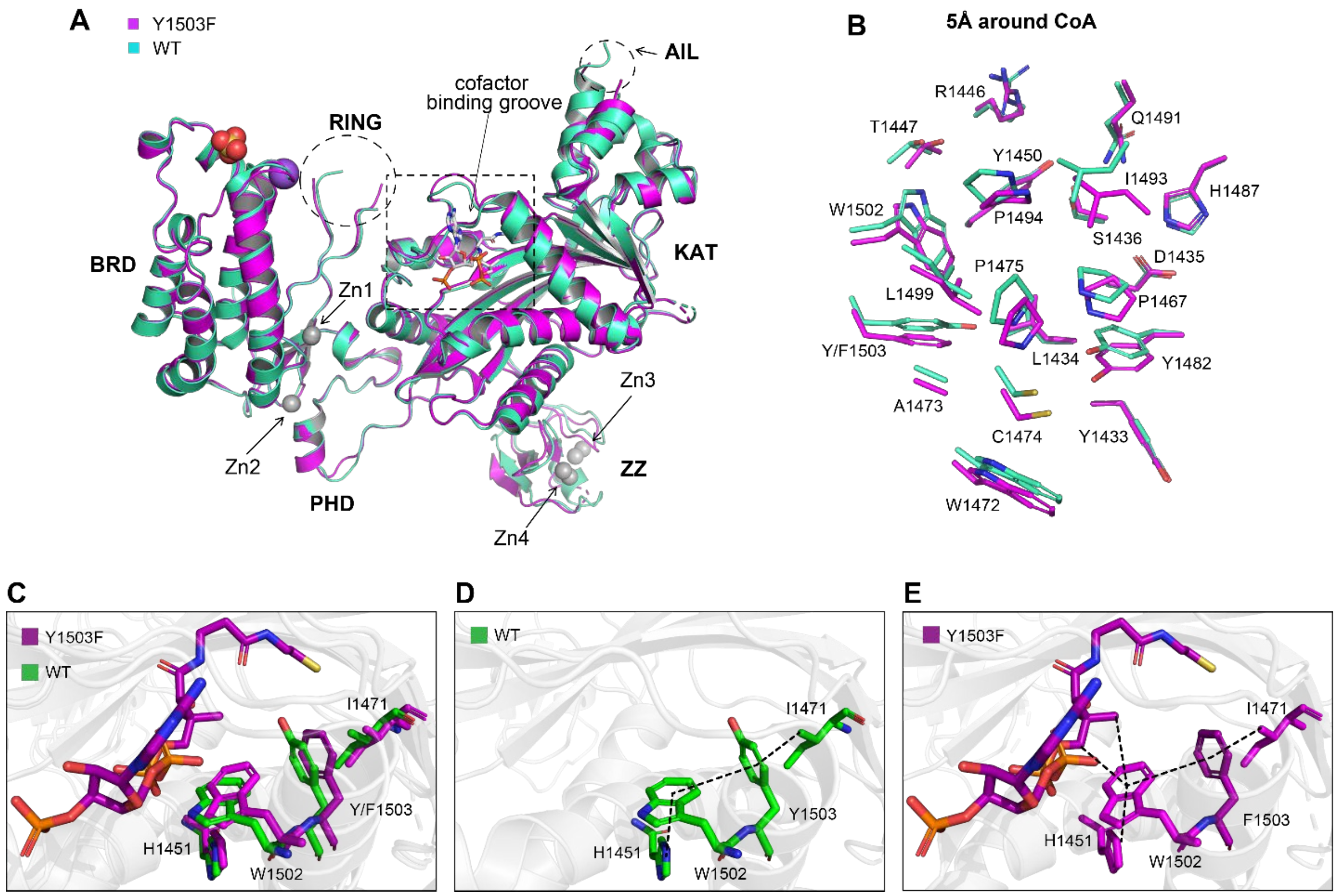

Fig. S4. Structural comparison of the CREBBP Y1503F variant with the apo wild-type (WT) structure. (A) Superposition of the CREBBP KAT structures of WT and Y1503F+CoA. The bromodomain, PHD and RING domains, and KAT cofactor-binding groove are indicated. (B) Superposition of residues within 5 Å of CoA in the Y1503F structure. (C-E) Close-up views of the CoA binding site in the WT and Y1503F structures, showing interactions between Y1503 (WT) or F1503 (Y1503F) and neighboring residues, as well as CoA. WT structure reference: PDB ID: 5U7G.

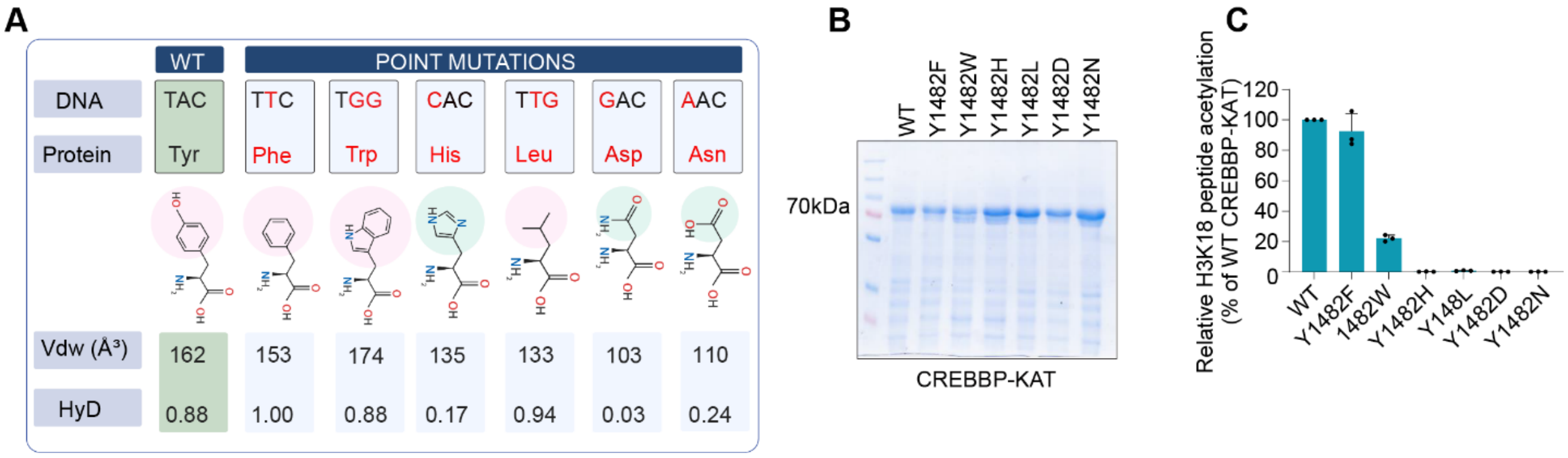

Fig. S5. Acetyltransferase activity of the CREBBP Y1482 variants. (A) Mutagenesis strategy for residue Y1482, showing corresponding codon changes, amino acid substitutions, and hydrophobicity. (B) Coomassie-stained SDS-PAGE of purified CREBBP-KAT Y1482 variants. (C) RP-UFLC-based acetyltransferase activity of CREBBP-KAT Y1482 variants using the H3K18 peptide, shown relative to WT.

A

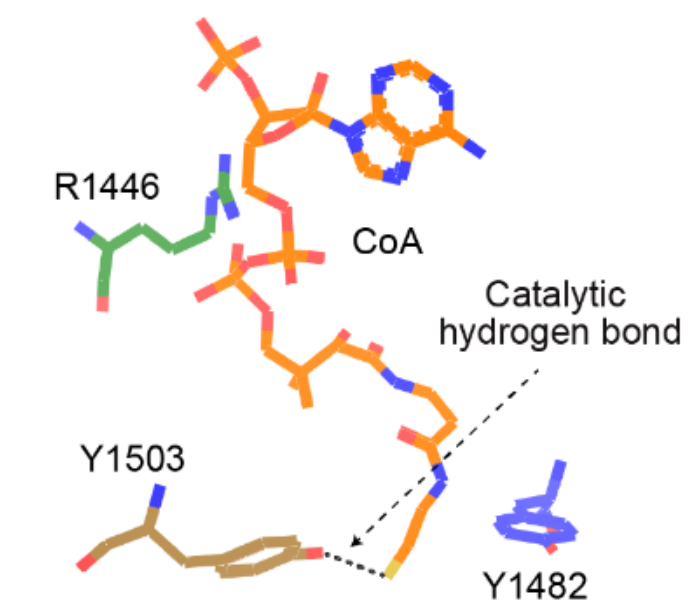

B

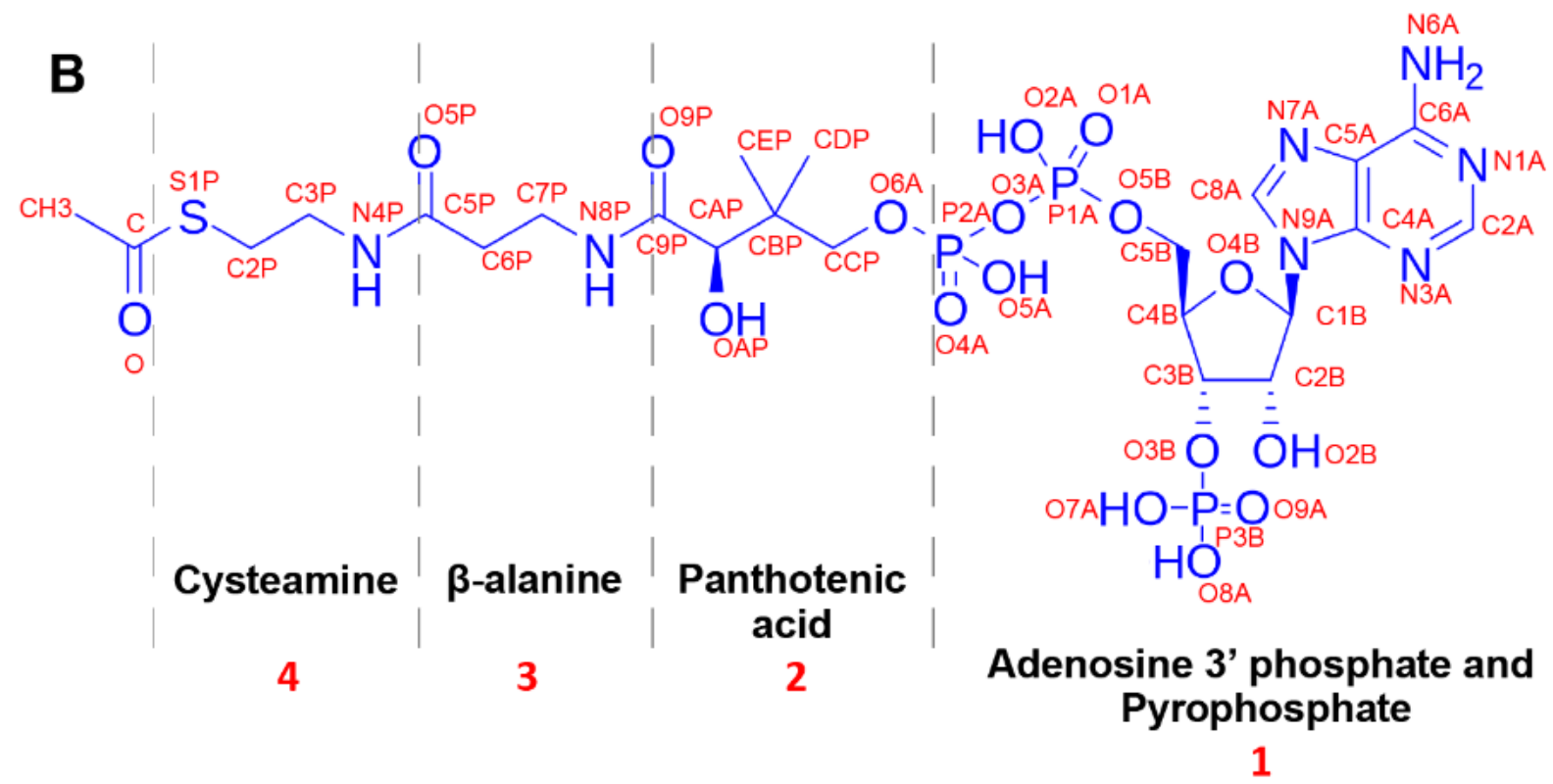

C

Residues	# of residue atoms interacting with CoA					# of Mutation		Roles of Residues
	S atom	CoA-4	CoA-3	CoA-2	CoA-1	Lymphomas	Solid	
F1503	2	2	0	0	0	93	1	Catalytic
W1472	1	1	0	0	0	15	2	
Y1482	0	5	1	0	0	44	3	Shaping catalytic center
L1434	0	3	2	0	0	6	0	
C1474	0	1	0	0	0	10	0	
P1476	0	1	3	0	0	9	2	
L1499	0	1	0	1	0	20	1	
D1435	0	0	1	0	0	38	3	
S1436	0	0	0	9	9	25	2	CoA binding stabilization
*W1502	0	0	0	4	2	42	3	
Y1450	0	0	0	3	1	33	2	
P1494	0	0	0	2	7	2	2	
Q1491	0	0	0	1	0	14	2	
R1446	0	0	0	1	9	190	20	
K1492	0	0	0	0	15	0	0	
K1495	0	0	0	0	8	1	1	
R1498	0	0	0	0	8	3	1	
I1493	0	0	0	0	6	5	0	
T1447	0	0	0	0	5	0	1	

D

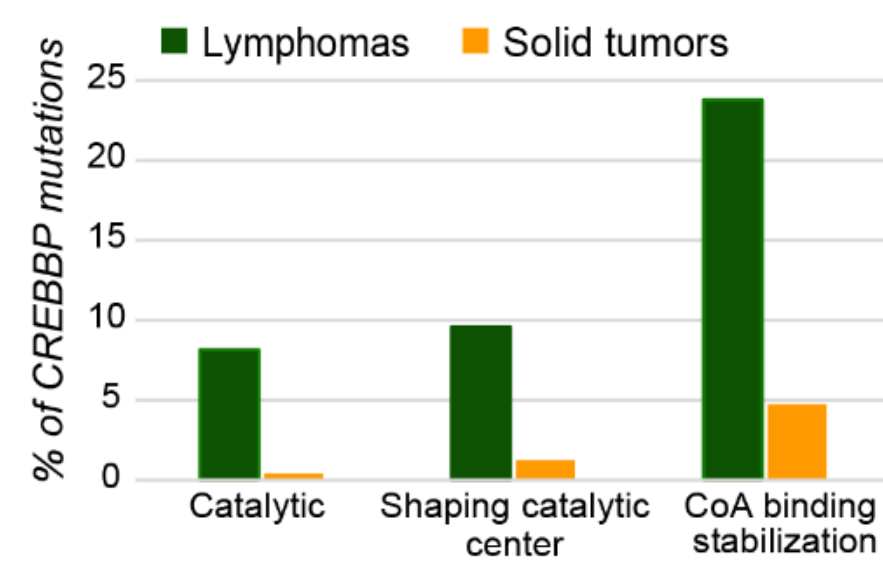

E

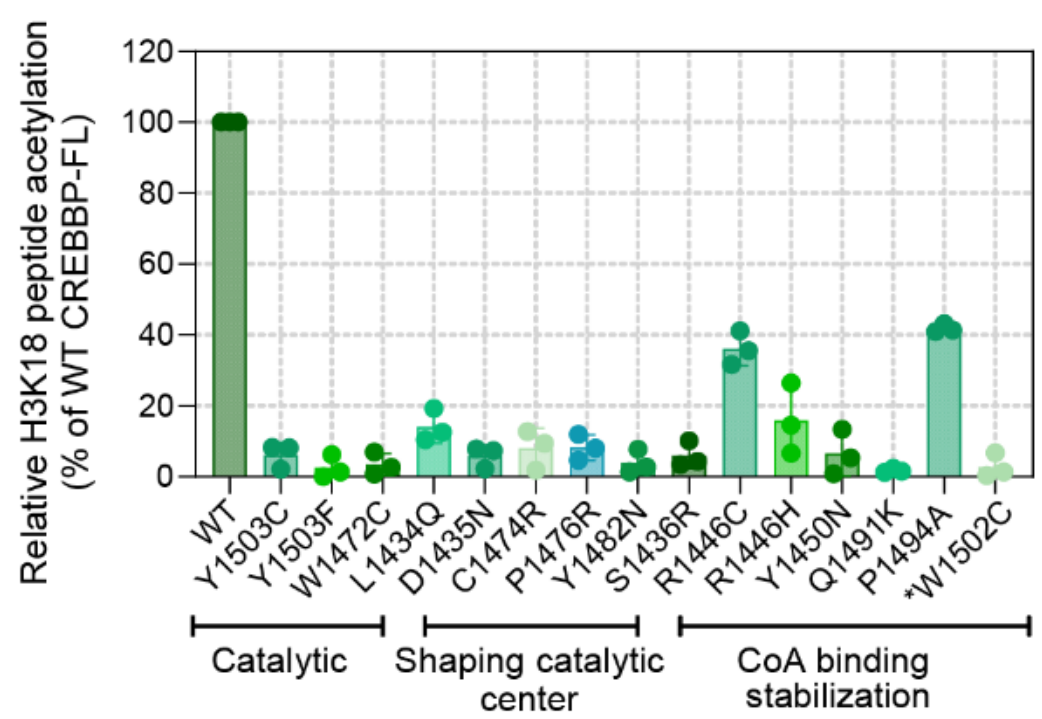

F

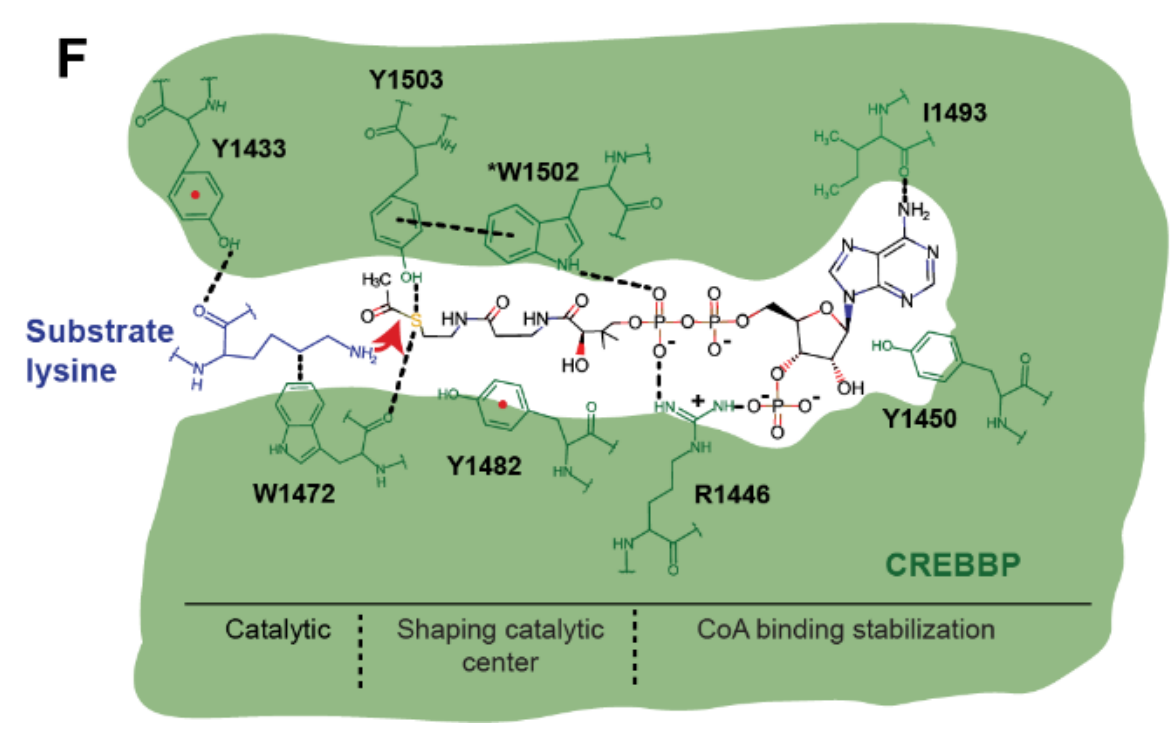

Fig. S6: Recurrent CREBBP mutations impair Ac-CoA engagement. (A) Structural view of the CREBBP CoA binding pocket showing the positions of R1446, Y1482, and Y1503 relative to CoA. (B) Schematic representation of the Ac-CoA molecule, with constituent chemical groups indicated. (C) Interactions between CoA and Y1503F at the atomic level. These interactions were identified using PDBsum (http://www.ebi.ac.uk/thornton-srv/databases/pdbsum/) based on the CREBBP Y1503F mutant structure (PDB ID: 8OG2). (D) Frequency of *CREBBP* mutations affecting distinct functional classes in lymphomas and solid tumors. (E) RP-UFLC-based acetyltransferase activity of CREBBP-FL WT and hotspot variants toward H3K18 peptide. CREBBP proteins were expressed in HEK293T cells, immunoprecipitated, and incubated with H3K18 peptide and Ac-CoA. RP-UFLC-quantified peptide acetylation was normalized to immunoprecipitated CREBBP protein levels and expressed relative to WT activity. (F) Model of the CREBBP active site illustrating interactions with Ac-CoA (black) and the substrate lysine (blue). Residues are grouped by functional role, including substrate-binding stabilization, shaping of the catalytic center, and Ac-CoA binding stabilization. Dashed lines indicate hydrogen-bond or ionic interactions; red dots indicate aromatic interactions. *Residue W1502 contributes to CoA binding stabilization through hydrogen bonding with the pyrophosphate moiety and also contributes to catalysis via π–π interactions with the catalytic residue Y1503.

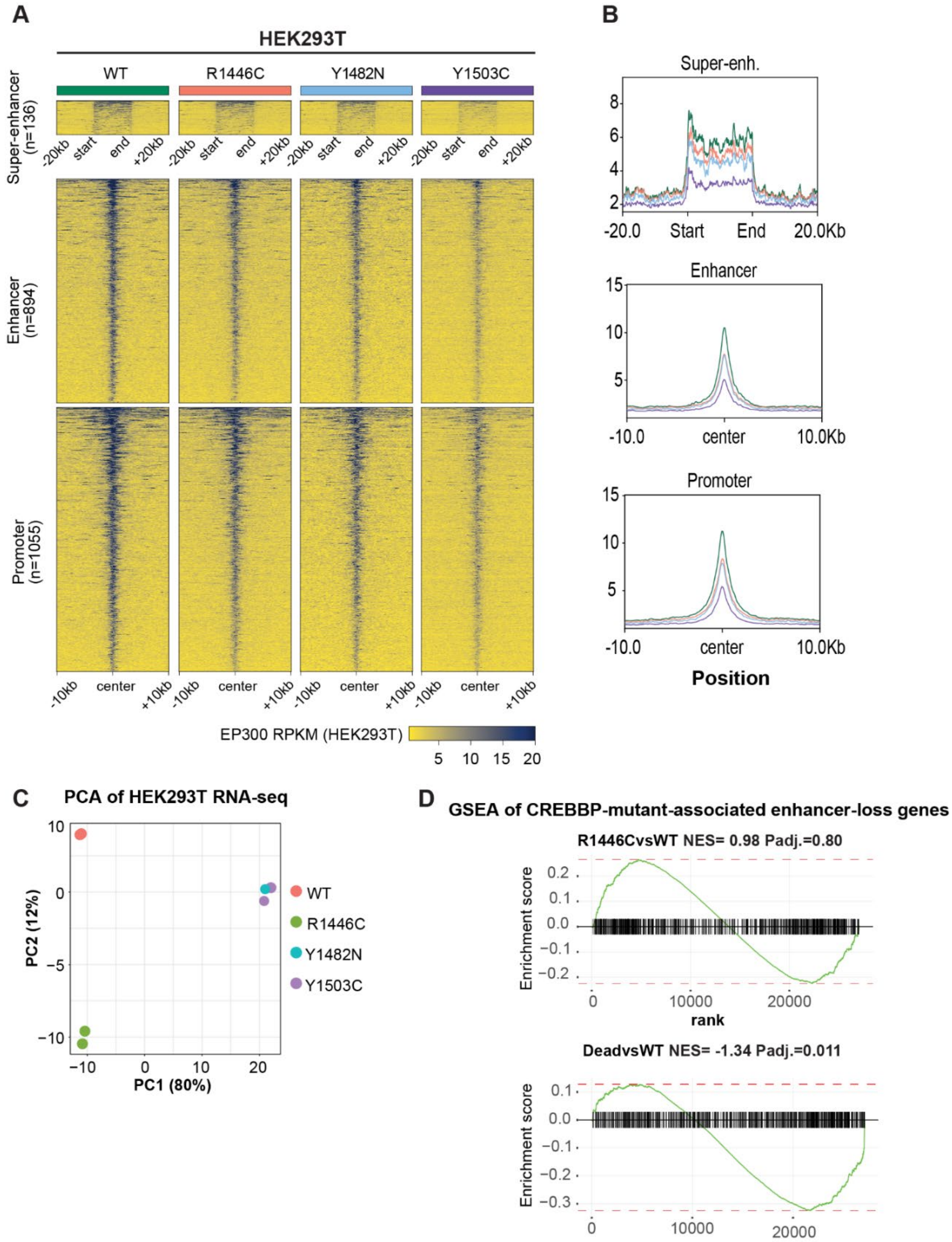

Fig. S7: CREBBP KAT domain hotspot mutations alter EP300 chromatin occupancy. (A) Heatmaps of EP300 CUT&RUN signal at super-enhancers, enhancers, and promoters in HEK293T cells expressing WT or mutant CREBBP (R1446C, Y1482N, or Y1503C). (B) Average EP300 CUT&RUN signal profiles at super-enhancers, enhancers, and promoters corresponding to the heatmaps. (C) Principal component analysis of RNA-seq data from HEK293T cells expressing WT or mutant CREBBP. (D) GSEA using the enhancer-

loss-associated gene set comparing R1446C or the catalytically dead CREBBP mutants (Y1482N or Y1503C) with WT cells.

Tables

Table S1: Data collection, phasing and refinement statistics.

	Y1503C/CoA	Y1482N/CoA	R1446C/CoA	Y1503F/CoA	R1446H/CoA
PDB ID	8CNB	8CN0	8CMZ	8OG2	9TZ6
Resolution range (Å)	53.7–2.0	107.3–2.4	105.7–2.3	106.2–2.5	77.3–2.5
Space group	I 2 2 2	I 2 2 2	I 2 2 2	I 2 2 2	I 2 2 2
Unit cell a, b, c (Å)	86.7, 144.8, 155.4	87.0, 148.7, 155.2	87.2, 145.1, 154.8	86.8, 145.7, 155.3	86.9, 146.8, 154.7
Unit cell α, β, γ (°)	90, 90, 90	90, 90, 90	90, 90, 90	90, 90, 90	90, 90, 90
Total reflections	433305 (19762)	235998 (10114)	316379 (14126)	268753 (11895)	178314 (8182)
Unique reflections	32743 (1638)	23400 (1171)	23434 (1172)	19830 (991)	13692 (686)
Multiplicity	13.2 (12.1)	10.1 (8.6)	13.5 (12.1)	13.6 (12.0)	13.0 (11.9)
Completeness (%)	89.8 (70.9)	94.5 (67.3)	94.2 (74.4)	93.5 (62.8)	89.8 (69.8)
Mean I/σ(I)	12.7 (2.0)	13.9 (1.6)	12.2 (1.9)	12.8 (1.6)	8.8 (1.6)
Rmerge	0.105 (1.241)	0.111 (1.386)	0.122 (1.383)	0.142 (1.756)	0.208 (1.687)
Rmeas	0.109 (1.294)	0.117 (1.473)	0.127 (1.442)	0.148 (1.832)	0.216 (1.763)
Rpim	0.030 (0.361)	0.037 (0.490)	0.034 (0.401)	0.040 (0.515)	0.060 (0.504)
CC1/2	0.999 (0.799)	0.998 (0.597)	0.998 (0.741)	0.998 (0.553)	0.998 (0.666)
**Refinement**					
Reflections used in refinement	32743	23400	23434	19830	13692
Reflections used for R-free	1693	1160	1173	1006	682
Rwork	0.262 (0.686)	0.235 (0.369)	0.239 (0.404)	0.236 (0.449)	0.249 (0.282)
Rfree	0.316 (0.572)	0.281 (0.263)	0.297 (0.501)	0.289 (0.441)	0.276 (0.334)
Number of non-hydrogen atoms	4514	4509	4481	4489	4479
macromolecules	4409	4426	4415	4414	4413
ligands	52	53	52	58	52
solvent	53	30	14	17	14
RMS bonds (Å)	0.013	0.014	0.011	0.011	0.009
RMS angles (°)	1.85	2.02	1.37	1.43	1.06
Ramachandran favored (%)	96.7	95.4	93.1	93.5	96.0
Ramachandran allowed (%)	2.9	4.0	6.9	6.1	3.8
Ramachandran outliers (%)	0.4	0.6	0.0	0.4	0.2
Rotamer outliers (%)	0.2	0.0	0.0	0.2	7.5
Clashscore	18.9	19.5	22.4	20.5	12.0
Average B-factor (Å²)	67.3	66.3	75.9	77.1	83.3
macromolecules	67.3	66.3	75.4	77.1	82.9
ligands	88.9	74.1	122.3	81.9	127.0
solvent	44.3	49.2	52.2	53.0	48.9

Statistics for the highest-resolution shell are shown in parentheses.

Supplementary References

1. X. Liu *et al.*, The structural basis of protein acetylation by the p300/CBP transcriptional coactivator. *Nature* **451**, 846-850 (2008).
2. H. Yang *et al.*, Blunted CD40-responsive enhancer activation in CREBBP-mutant lymphomas can be restored by enforced CD4 T-cell engagement. *Blood* **146**, 191-205 (2025).
